# Salinity Tolerance and Antioxidant Response in Watermelon Seedlings Pre-Treated with Abiotic Stress Attenuators

**DOI:** 10.3390/plants15081227

**Published:** 2026-04-16

**Authors:** Moadir de Sousa Leite, Salvador Barros Torres, Clarisse Pereira Benedito, Kleane Targino Oliveira Pereira, Maria Valdiglezia de Mesquita Arruda, Jéssica Christie Dantas de Oliveira Costa, Giovanna Dias de Sousa, Angie Alejandra Rodriguez Cruz, João Pedro Gonçalves Bispo, Charline Zaratin Alves, Pablo Ferreira da Silva, Marco Porceddu, Gianluigi Bacchetta, Alex Álvares da Silva, Francisco Vanies da Silva Sá

**Affiliations:** 1 Department of Agricultural and Forestry Sciences, Federal Rural University of the Semi-Arid, Mossoró 59625-900, RN, Brazil; moadir@outlook.com (M.d.S.L.); sbtorres@ufersa.edu.br (S.B.T.); clarisse@ufersa.edu.br (C.P.B.); kleane_rn@hotmail.com (K.T.O.P.); jessicachristie73@gmail.com (J.C.D.d.O.C.); giodiassousa@hotmail.com (G.D.d.S.); pablofesilva@gmail.com (P.F.d.S.); 2 Postgraduate Program in Soil and Water Management, Federal Rural University of the Semi-Arid, Mossoró 59625-900, RN, Brazil; valdigleziaarruda@yahoo.com.br; 3 Postgraduate Program in Phytotechnics, University of the Semi-Arid—UFERSA, Mossoró 59625-900, RN, Brazil; angie.cruz@alunos.ufersa.edu.br; 4 Department Agricultural Sciences, Federal University of Mato Grosso do Sul, Chapadão do Sul 79560-000, MS, Brazil; joao22p3dro@gmail.com (J.P.G.B.); charline.alves@ufms.br (C.Z.A.); 5 Centre for Conservation of Biodiversity (CCB), Department of Life and Environmental Sciences, University of Cagliari, 09123 Cagliari, Italy; porceddu.marco@unica.it (M.P.); gianluigi.bacchetta@unica.it (G.B.); 6 Department of Agrarian and Exact Sciences, Universidade Estadual da Paraíba, Sítio Cajueiro, Catolé Do Rocha 54888-000, PB, Brazil; alex.alvares@visitante.uepb.edu.br

**Keywords:** *Citrullus lanatus*, Cucurbitaceae, abiotic stress, oxidative stress, salt stress

## Abstract

Salinization of agricultural areas is one of the main abiotic factors responsible for the reduction of seed germination and vigor. In this context, the use of stress attenuators applied to seeds may contribute to mitigating the effects of salinity and improving the physiological and antioxidant performance of seedlings. This study aimed to evaluate the effects of stress attenuators on the tolerance and antioxidant activity of watermelon *Citrullus lanatus* (Thunb.) Matsum & Nakai cultivars under saline conditions. The study was conducted in two stages. In the first stage, a 3 × 6 factorial scheme was used to evaluate three salinity levels (0, −0.2, and −0.4 MPa) and six watermelon cultivars. In the second stage, in a 2 × 6 factorial scheme, two cultivars (sensitive and tolerant) were subjected to the combination of salinity (−0.4 MPa) and attenuators: hydropriming, gibberellic acid, salicylic acid, and hydrogen peroxide. Physiological and biochemical traits were evaluated, including hydrogen peroxide content, lipid peroxidation, and the activity of the enzymes, superoxide dismutase, catalase, and ascorbate peroxidase. Salinity reduced germination and seedling vigor, with Crimson Sweet, Charleston Gray, and Charleston Super being the most sensitive cultivars, whereas Congo and Omaru exhibited greater tolerance, and Fairfax also showed good performance under saline conditions. The selection of cultivars for the second stage was based not only on physiological tolerance but also on agronomic and commercial relevance, including post-harvest resistance traits. Seed treatment of Crimson Sweet with salicylic acid and hydrogen peroxide increased antioxidant enzyme activity, with increases of up to 103% in ascorbate peroxidase activity, and reduced oxidative damage, with reductions of 44% in hydrogen peroxide and 49% in malondialdehyde levels. In Fairfax, gibberellic acid contributed to osmotic adjustment, promoting increase of up to 76% in total soluble sugars, while pre-germinative treatment with salicylic acid and hydrogen peroxide promoted higher enzyme activity, contributing to the reduction of oxidative stress.

## 1. Introduction

Watermelon [*Citrullus lanatus* (Thunb.) Matsum & Nakai], belonging to the family Cucurbitaceae, plays an important role in human nutrition, particularly in tropical regions where its consumption is highly significant [1]. However, the salinization of agricultural lands has, for decades, become an increasing problem in several regions of the world, with salinity stress representing one of the main abiotic factors responsible for reducing crop productivity [2]. Watermelon is considered a salt-sensitive species and, when continuously exposed to saline conditions, may exhibit irreversible damage, especially during the early stages of seedling development [3].

In saline substrates, water uptake by seeds is reduced due to the decrease in the water potential gradient between the seed and the surrounding medium. This condition compromises imbibition impairs reserve mobilization and may cause disturbances in the membrane system of the embryonic axis [4]. In addition, excessive salt concentrations can induce toxic effects due to ion accumulation, affecting enzymatic activities essential for the germination process and resulting in a longer time for radicle protrusion and seedling establishment [5]. At the physiological level, salinity stress is commonly divided into two phases: an initial osmotic phase, which limits water absorption and cell expansion, and a subsequent ionic phase, characterized by the accumulation of Na^+^ and Cl^−^ ions in plant tissues. This ionic imbalance may lead to nutrient deficiency, particularly of K^+^ and Ca^2+^, disruption of metabolic processes, and impairment of photosynthetic efficiency. Furthermore, salinity induces the overproduction of reactive oxygen species (ROS), resulting in oxidative stress, lipid peroxidation, and damage to proteins and nucleic acids [2,3,5].

From an agronomic perspective, these physiological disturbances directly affect crop establishment, reducing seedling uniformity, delaying emergence, and ultimately compromising yield and fruit quality. In watermelon, these effects are particularly critical during early developmental stages, as poor seedling establishment may limit stand formation and reduce the productive potential of the crop in saline environments.

In this context, studies addressing plant tolerance to salinity stress involve the investigation of different molecular, physiological, and morphological mechanisms, as well as the development of techniques capable of increasing plant resistance to this adverse condition [6]. Among these techniques, seed hydropriming has been widely investigated in horticultural species [7]. According to these authors, the procedure consists of controlled seed imbibition, which can induce protection mechanisms, cellular repair, and metabolic protection, thereby favoring seed acclimatization and increasing their capacity to tolerate subsequent environmental stresses.

Among the substances used to mitigate the effects of salinity stress, gibberellic acid stands out due to its role in enzymatic metabolism, promoting the induction and acceleration of the germination process and favoring the initial development of seedlings [8]. Similarly, salicylic acid has been widely recognized as an important modulator of plant defense responses, acting in the activation of the antioxidant system and in increasing the activity of enzymes such as peroxidases, superoxide dismutase, and catalase [6]. In addition to these substances, hydrogen peroxide may also act as a stress attenuator, functioning as a signaling molecule capable of regulating hormonal interactions, particularly between abscisic acid and gibberellins, and triggering intracellular responses associated with tolerance to salinity stress [9].

Among the main plant defense mechanisms against salinity stress, the enzymatic antioxidant system stands out, primarily composed of the enzymes superoxide dismutase (SOD), catalase (CAT), and ascorbate peroxidase (APX) [10,11]. Increased activity of these enzymes represents an important defense strategy, as it enables the elimination of reactive oxygen species (ROS) that accumulate during plant exposure to stress conditions, thereby contributing to the maintenance of cellular redox balance and reducing oxidative damage that may impair plant growth and development [12].

In this context, seed treatment with substances capable of inducing physiological and biochemical mechanisms associated with salinity tolerance becomes a promising strategy to promote the initial establishment of plants in saline environments, contributing to food production and agricultural sustainability, particularly in regions subject to this type of stress.

The hypothesis of this study is that the application of stress-attenuating substances via seed treatment can mitigate the adverse effects of salinity by stimulating the antioxidant defense system of plants, with the intensity of the response being influenced by the level of cultivar tolerance to stress. Therefore, the objective of this study was to evaluate the tolerance of watermelon cultivars and the effects of stress attenuators on the antioxidant activity and physiological performance of watermelon seeds and seedlings subjected to salinity.

## 2. Results

### 2.1. Stage I—Selection of Watermelon Cultivars and Determination of the Saline Stress Level to Be Used in Stage II

The analysis of variance revealed a significant interaction between the factors (cultivars × salinity levels) for all variables evaluated in the first stage of the experiment, except for shoot length. This indicates that the cultivars responded differently under saline conditions, with responses varying according to the salt concentration in the substrate (Table 1).

Salinity reduced germination and the initial performance of watermelon seedlings, with responses varying among cultivars (Table 1). Germination remained high in most cultivars at −0.2 MPa; however, more pronounced reductions were observed at −0.4 MPa, especially for Charleston Super (60%), Charleston Gray (74%), and Crimson Sweet (76%). In contrast, Fairfax showed greater stability, maintaining 95% germination at the highest salinity level. The germination speed index also decreased with increasing salinity in all cultivars. Even under −0.4 MPa, Fairfax presented the highest GSI values (14.9), whereas Crimson Sweet and Charleston Gray exhibited the greatest reductions (Table 1).

**Figure 1:**
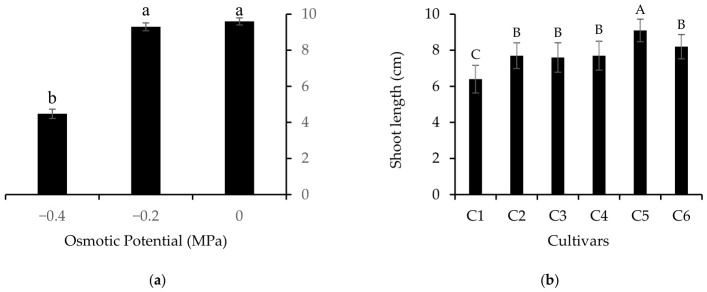
Shoot length of watermelon (*Citrullus lanatus* (Thunb.) Matsum. & Nakai) seedlings as a function of different salinity levels (**a**) and cultivars (**b**). C1 = Crimson Sweet; C2 = Fairfax; C3 = Charleston Gray; C4 = Charleston Super; C5 = Omaru; C6 = Congo. Means followed by the same lowercase letter (osmotic potential) and uppercase letter (cultivars) do not differ according to the Scott–Knott test at 5% probability.

Shoot length was affected by the factors independently, with no interaction between them (Figure 1). Regarding osmotic potential (Figure 1a), the level of −0.4 MPa resulted in a significant reduction in this variable. Among the cultivars (Figure 1b), differences in shoot growth were observed. The cultivar Omaru (C5) presented the greatest shoot length, differing from the others. Fairfax (C2), Charleston Gray (C3), Charleston Super (C4), and Congo (C6) showed intermediate values, while Crimson Sweet (C1) presented the lowest shoot length.

Root length was reduced under higher salinity, with more pronounced reductions in Crimson Sweet and Charleston Super. In contrast, Fairfax, Omaru, and Congo maintained greater root lengths under saline stress (Table 1). Regarding biomass variables, Fairfax showed the highest shoot and root dry mass values at all salinity levels, indicating greater seedling vigor. More pronounced reductions were observed in Crimson Sweet and Charleston Super.

Increasing salinity promoted an increase in the contents of total soluble sugars, free amino acids, and proline in all cultivars. The highest accumulations were observed at −0.4 MPa, particularly in Charleston Gray, which showed the highest levels of free amino acids (100.0 µmol g^−1^ FM) and proline (3.3 µmol g^−1^ FM). Overall, Fairfax, Omaru, and Congo showed better physiological and growth performance under saline conditions, whereas Crimson Sweet and Charleston Super exhibited greater sensitivity to stress.

For the selection of the salinity level to be used in the second stage of the experiment, the reduction in germination and seedling performance variables was primarily considered. Therefore, the level of −0.4 MPa was adopted for the second stage, as it could cause detrimental effects in both sensitive and tolerant cultivars. Moreover, the use of stress attenuators may allow partial or complete recovery of physiological variables in the second stage.

**Figure 2:**
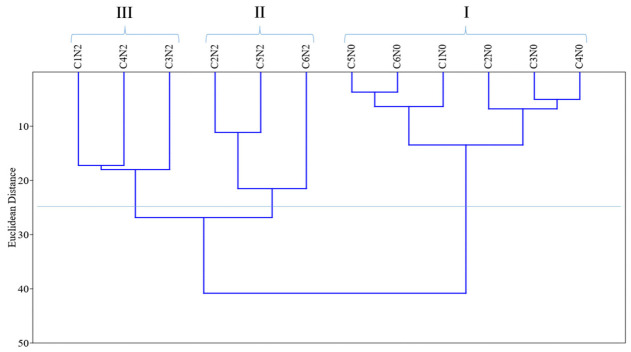
Dendrogram of dissimilarity of the groups formed by the combination of salinity levels (N) and watermelon (*Citrullus lanatus* (Thunb.) Matsum. & Nakai) cultivars (C). N0 = Control; N2 = −0.4 MPa; C1 = Crimson Sweet; C2 = Fairfax; C3 = Charleston Gray; C4 = Charleston Super; C5 = Omaru; C6 = Congo.

Cluster analysis (Figure 2) was performed using Euclidean distance as the dissimilarity measure, with a cutoff distance of 25.0 to form groups based on combinations between osmotic potential levels (N) and watermelon cultivars (C). Group I was characterized by the six cultivars under the control condition (0.0 MPa), with no salinity. Group II comprised the cultivars Congo, Omaru, and Fairfax, which stood out with higher germination and superior performance in biochemical and growth variables under the highest salinity level (−0.4 MPa), appearing in the dendrogram close to the control group. In contrast, Group III consisted of the cultivars most sensitive to salinity, with Crimson Sweet identified as the most susceptible, due to its position at the far left of the dendrogram.

Data analysis allowed the differentiation of cultivars according to their tolerance to salinity levels. Crimson Sweet showed the lowest tolerance to salinity and was therefore selected for the second stage. The cultivars Congo, Omaru, and Fairfax showed greater tolerance to salinity, with Fairfax selected for the second stage. The selection of cultivars also considered market demand, as Crimson Sweet and Fairfax are widely cultivated and commercially important, which further supported their inclusion in the second stage.

Thus, the salinity level of −0.4 MPa was defined for use in the second stage of the evaluations, and the cultivars Fairfax and Crimson Sweet were selected as tolerant and sensitive to salinity, respectively. Although Fairfax was not identified as the most tolerant cultivar, it was selected due to its market relevance and postharvest resistance characteristics.

### 2.2. Stage II—Salt Stress Attenuators and Watermelon Defense System

The analysis of variance revealed isolated effects of cultivars and treatments on germination, germination speed index, and shoot and root dry mass. However, for shoot and root length, total soluble sugars, amino acids, free proline, hydrogen peroxide, lipid peroxidation expressed as malondialdehyde (MDA) content, and the activity of the enzymes superoxide dismutase (SOD), catalase (CAT), and ascorbate peroxidase (APX), a significant interaction between factors was observed. This indicates that, for these variables, the responses to treatments depended on the cultivar’s tolerance to salinity.

**Figure 3:**
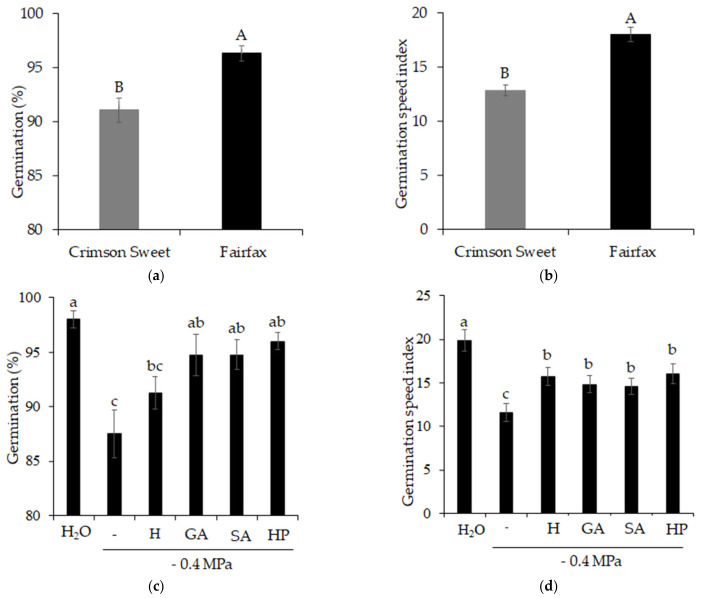
Germination (**a**,**c**) and germination speed index (**b**,**d**) of watermelon (*Citrullus lanatus* (Thunb.) Matsum. & Nakai) cultivars subjected to pre-germination treatments and salinity. Control (H_2_O); salinity without attenuators (−0.4 MPa); salinity + hydropriming (H); salinity + gibberellic acid (GA); salinity + salicylic acid (SA); salinity + hydrogen peroxide (HP). Means followed by the same uppercase letter (cultivars) and lowercase letter (pre-germination treatments) do not differ according to Student’s *t*-test and Tukey’s test, respectively, at the 5% probability level.

Germination (Figure 3a) and germination speed index (Figure 3b) of the cultivars Crimson Sweet and Fairfax differed significantly according to their degree of salinity tolerance. Germination and germination speed values of the cultivar Fairfax were 6 and 40% higher, respectively, than those of Crimson Sweet.

Regarding the response of germination (Figure 3c) and germination speed index (Figure 3d) to treatments, salinity (−0.4 MPa) was detrimental to these variables, reducing germination and germination speed by 11 and 42%, respectively, compared with the control treatment. However, beneficial effects on both variables were observed with the use of seed treatments with attenuators. Seeds treated with gibberellic acid (GA), salicylic acid (SA), and hydrogen peroxide (HP) showed germination values like the control. For the germination speed index, although the values observed in the control were not reached, pre-germination treatments with hydropriming (H), GA, SA, and HP increased germination speed by 36, 28, 26, and 39%, respectively, compared with salinity.

**Figure 4:**
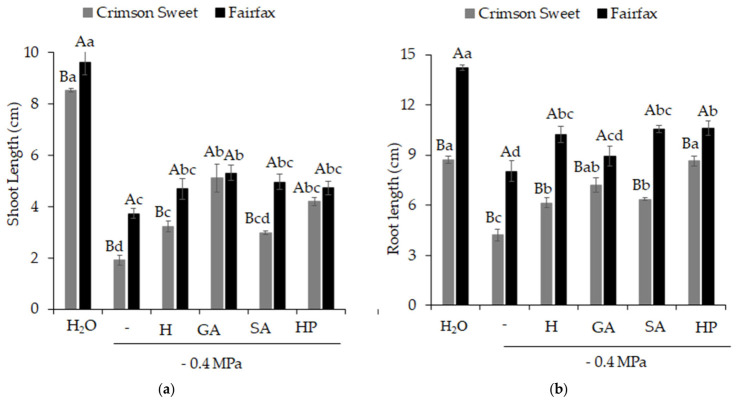
Shoot length (**a**) and root length (**b**) of watermelon (*Citrullus lanatus* (Thunb.) Matsum. & Nakai) cultivars are subjected to pre-germination treatments and salinity. Control (H_2_O); salinity without attenuators (−0.4 MPa); salinity + hydropriming (H); salinity + gibberellic acid (GA); salinity + salicylic acid (SA); salinity + hydrogen peroxide (HP). Means followed by the same uppercase letter (cultivars) and lowercase letter (pre-germination treatments) do not differ according to Student’s *t*-test and Tukey’s test, respectively, at the 5% probability level.

Shoot (Figure 4a) and root (Figure 4b) lengths of both cultivars were negatively affected by salinity. However, the use of attenuators was beneficial to both shoot and root growth of watermelon seedlings, resulting in greater values compared with salinity. For both cultivars, shoot length resulting from the pre-germination treatment with GA produced the best results, being 167 and 43% higher than salinity for the cultivars Crimson Sweet and Fairfax, respectively. For root length, hydropriming (H), SA, and HP resulted in greater values than salinity in the cultivar Fairfax, whereas treatments with GA and HP produced root lengths statistically like those obtained in the control for Crimson Sweet.

**Figure 5:**
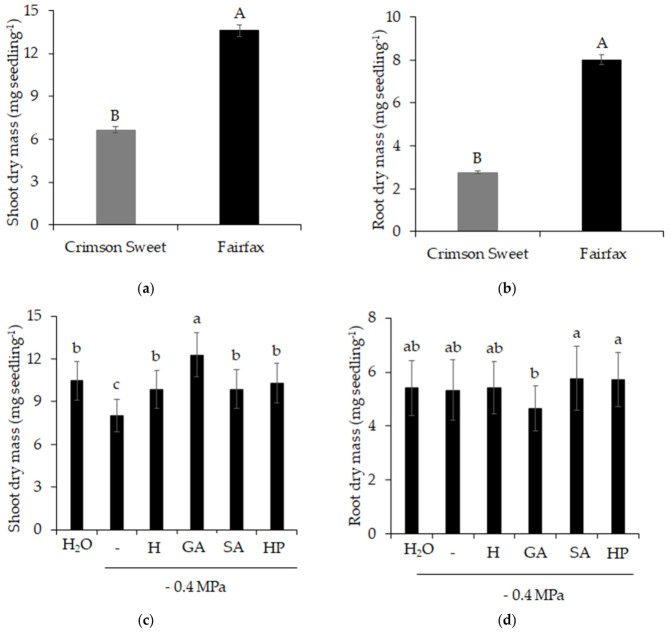
Shoot dry mass (**a**,**c**) and root dry mass (**b**,**d**) of watermelon (*Citrullus lanatus* (Thunb.) Matsum. & Nakai) cultivars subjected to pre-germination treatments and salinity. Control (H_2_O); salinity without attenuators (−0.4 Mpa); salinity + hydropriming (H); salinity + gibberellic acid (GA); salinity + salicylic acid (SA); salinity + hydrogen peroxide (HP). Means followed by the same uppercase letter (cultivars) and lowercase letter (pre-germination treatments) do not differ according to Student’s *t*-test and Tukey’s test, respectively, at the 5% probability level.

The Shoot (Figure 5a) and root (Figure 5b) dry masses of the cultivar Fairfax were 104 and 191% higher than those obtained for Crimson Sweet. Regarding treatments, shoot dry mass was favored using pre-germination treatments, particularly GA, which resulted in an accumulation 53% higher than that observed under salinity (Figure 5c). In contrast, the use of GA resulted in lower accumulation of root dry mass, although it did not differ statistically from salinity or control (Figure 5d).

**Figure 6:**
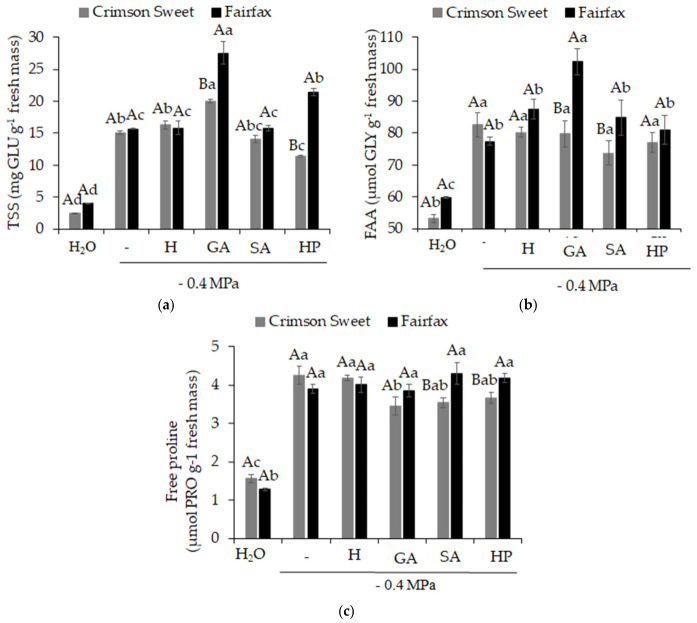
Total soluble sugars (**a**), free amino acids (**b**), and free proline (**c**) in watermelon (*Citrullus lanatus* (Thunb.) Matsum. & Nakai) cultivars subjected to pre-germination treatments and salinity. Control (H_2_O); salinity without attenuators (−0.4 MPa); salinity + hydropriming (H); salinity + gibberellic acid (GA); salinity + salicylic acid (SA); salinity + hydrogen peroxide (HP). Means followed by the same uppercase letter (cultivars) and lowercase letter (pre-germination treatments) do not differ according to Student’s *t*-test and Tukey’s test, respectively, at the 5% probability level.

In general, salinity promoted an increase in the concentration of compatible solutes in watermelon seedlings of both cultivars, with increases in the concentrations of total soluble sugars (Figure 6a), free amino acids (Figure 6b), and free proline (Figure 6c). For the Crimson Sweet, no benefits were observed from the use of pre-germination treatments in terms of inducing solute accumulation, except for GA, which promoted total soluble sugar accumulation 33% higher than that observed under salinity. GA also produced the best results for Fairfax, as although it did not influence proline accumulation, its use resulted in greater accumulation of total soluble sugars and free amino acids, being 76 and 32% higher, respectively, than under salinity.

**Figure 7:**
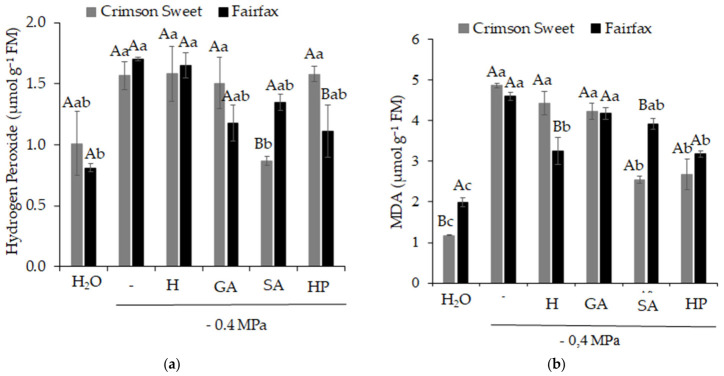
Hydrogen peroxide content (**a**) and malondialdehyde (**b**) in watermelon (*Citrullus lanatus* (Thunb.) Matsum. & Nakai) cultivars subjected to pre-germination treatments and salinity. Control (H_2_O); salinity without attenuators (−0,4 MPa); salinity + hydropriming (H); salinity + gibberellic acid (GA); salinity + salicylic acid (SA); salinity + hydrogen peroxide (HP). Means followed by the same uppercase letter (cultivars) and lowercase letter (pre-germination treatments) do not differ according to Student’s *t*-test and Tukey’s test, respectively, at the 5% probability level.

Hydrogen peroxide content (Figure 7a) and lipid peroxidation, expressed as MDA content (Figure 7b), increased significantly because of salinity, indicating the occurrence of stress caused by the presence of salt in the substrate. For the cultivar Crimson Sweet, a 44% reduction in hydrogen peroxide concentration was observed with the use of SA, while reductions of 49 and 45% in MDA were observed with SA and HP, respectively. For the cultivar Fairfax, all attenuators produced positive results, except for hydrogen peroxide under hydropriming (H) and MDA under GA and SA treatments.

**Figure 8:**
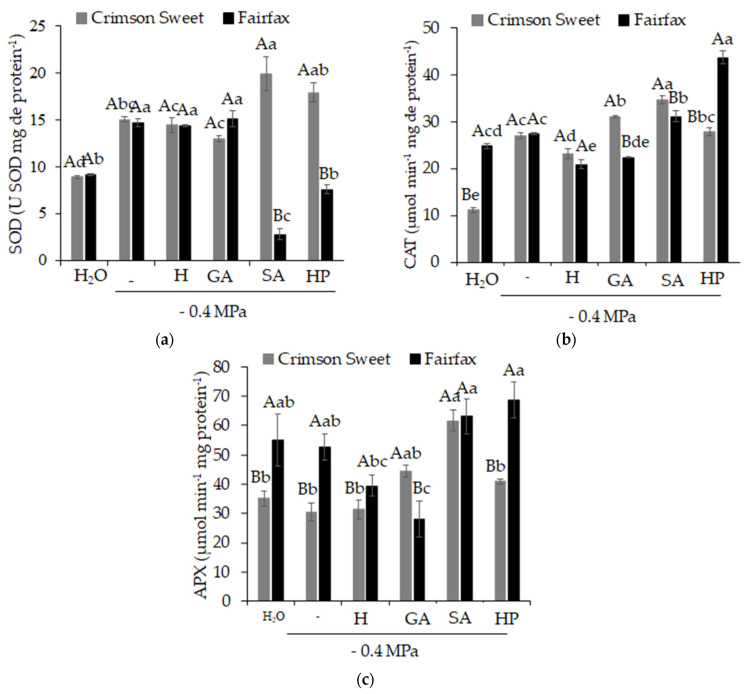
Activity of the enzymes superoxide dismutase—SOD (**a**), catalase—CAT (**b**), and ascorbate peroxidase—APX (**c**) in watermelon (*Citrullus lanatus* (Thunb.) Matsum. & Nakai) cultivars subjected to pre-germination treatments and salinity. Control (H_2_O); salinity without attenuators (−0.4 MPa); salinity + hydropriming (H); salinity + gibberellic acid (GA); salinity + salicylic acid (SA); salinity + hydrogen peroxide (HP). Means followed by the same uppercase letter (cultivars) and lowercase letter (pre-germination treatments) do not differ according to Student’s *t*-test and Tukey’s test, respectively, at the 5% probability level.

The results obtained for SOD (Figure 8a), CAT (Figure 8b), and APX (Figure 8c) demonstrate the influence of salinity and attenuators on the activity of these enzymes. For the cultivar Crimson Sweet, the pre-germination treatment of seeds with SA resulted in higher enzymatic activity for all enzymes evaluated, being 33, 29, and 103% higher than the activity observed under salinity for SOD, CAT, and APX, respectively. For this same cultivar, beneficial effects were also observed with the use of HP (SOD) and GA (CAT and APX), actively contributing to the mitigation of salinity effects.

**Figure 9:**
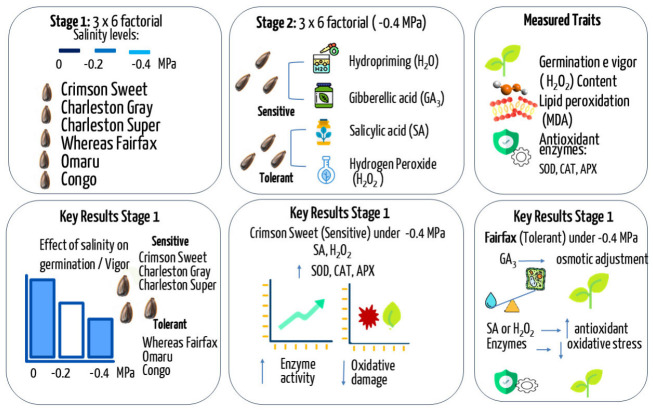
Overview of the experimental design and main outcomes of the two-stage study. In Stage 1, a 3 × 6 factorial design was used to evaluate the effects of three salinity levels (0, −0.2, and −0.4 MPa) on germination and vigor of six genotypes, classified as sensitive (Crimson Sweet, Charleston Gray, Charleston Super) and tolerant (Whereas Fairfax, Omaru, Congo). In Stage 2, conducted under −0.4 MPa, selected genotypes were subjected to priming treatments (hydropriming, gibberellic acid, salicylic acid, and hydrogen peroxide). The evaluated traits included germination and vigor, hydrogen peroxide (H_2_O_2_) content, lipid peroxidation (MDA), and antioxidant enzyme activities (SOD, CAT, and APX).

As shown in Figure 9, salinity negatively affects seed performance, while priming treatments contribute to improved antioxidant responses and reduced oxidative damage, with distinct responses between sensitive and tolerant genotypes.

## 3. Discussion

### 3.1. Stage I—Selection of Watermelon Cultivars and Determination of the Saline Stress Level to Be Used in Stage II

The reduction in germination and seedling vigor under higher salinity conditions indicates that salt stress compromises essential physiological processes during the early stage of watermelon development. The presence of salt in the substrate reduces the osmotic potential of the medium, hindering water absorption by seeds and delaying the onset of metabolic processes involved in germination [2]. This osmotic effect is often accompanied by ionic toxicity caused by the accumulation of Na^+^ and Cl^−^ ions, which may interfere with cellular metabolism and membrane integrity, thereby reducing seedling vigor [13].

The reduction observed in the germination speed index with increasing salinity confirms that salt stress not only decreases the final germination percentage but also delays the germination process. The decrease in GSI is frequently reported as one of the earliest indicators of salt stress in seeds, since the reduction in the osmotic potential of the medium limits water uptake and compromises essential metabolic processes for germination, including reserve mobilization and the enzymatic activity required for radicle protrusion [14].

The differences observed among cultivars indicate the existence of genetic variability in response to salt stress. Cultivars such as Fairfax, Omaru, and Congo exhibited a greater capacity to maintain germination and initial growth under higher salinity, suggesting greater physiological tolerance to stress. Variability in tolerance among watermelon genotypes has already been reported in previous studies, in which differences in early growth and germination were attributed to distinct capacities for osmotic regulation and maintenance of ionic homeostasis among cultivars [3].

The reduction in root growth under higher salinity further reinforces the negative effects of stress on seedling development. The root system is highly sensitive to salt accumulation, and reduced root growth may be associated with limitations in cell division and expansion caused by osmotic imbalance and ionic toxicity [13]. However, some cultivars were able to maintain greater root growth under stress, which may favor substrate exploration and contribute to increased tolerance to salinity.

The lower biomass accumulation observed under saline conditions is also associated with limitations imposed on cellular growth and plant energy metabolism. Salt stress may reduce photosynthetic efficiency and alter assimilate distribution, compromising dry matter accumulation in seedlings [2]. In this context, cultivars that maintain greater biomass production under stress exhibit greater tolerance potential and improved initial establishment.

The increase in total soluble sugars, free amino acids, and proline content observed with increasing salinity indicates the activation of osmotic adjustment mechanisms in seedlings. These compounds act as compatible osmolytes, contributing to the maintenance of cellular water potential and the protection of cellular structures under stress conditions [15]. Proline plays an important role in stabilizing proteins and membranes and acts in the scavenging of reactive oxygen species, assisting in the maintenance of cellular metabolism under adverse environments [16,17].

The greater accumulation of these compounds in certain cultivars may indicate a higher biochemical response capacity to salt stress, which contributes to the maintenance of seedling growth and development. In general, more tolerant plants tend to exhibit greater efficiency in activating antioxidant mechanisms and accumulating osmolytes, thereby reducing damage caused by osmotic and oxidative stress [10,11].

Cluster analysis allowed the identification of similarity patterns among combinations of cultivars and salinity levels, highlighting the separation between more tolerant and more sensitive genotypes. The proximity of the cultivars Fairfax, Omaru, and Congo to the control group indicates greater physiological stability under saline conditions, whereas the greater distance observed for Crimson Sweet reflects higher sensitivity to stress. This type of multivariate approach has been widely used to identify genotypes with greater potential for adaptation to saline environments, allowing the selection of more promising materials for breeding programs or for subsequent experimental stages [3].

Based on these results, the selection of an osmotic potential of −0.4 MPa for the second stage of the experiment proved appropriate, as this level was sufficient to impose stress capable of differentiating cultivars in terms of salinity tolerance. The selection of the cultivars Fairfax and Crimson Sweet as contrasting materials is also consistent with the results obtained, allowing a clearer evaluation of the effects of salt stress mitigation strategies in the subsequent stages of the study.

### 3.2. Stage II—Salt Stress Attenuators and Watermelon Defense System

The negative effects of salinity on germination and early seedling growth are mainly associated with the osmotic and ionic components of salt stress. Initially, the reduction in the osmotic potential of the medium hinders water uptake by seeds, compromising imbibition and delaying the metabolic processes involved in germination. At later stages, the accumulation of toxic ions such as Na^+^ and Cl^−^ may cause nutritional imbalance, metabolic alterations, and cellular damage [10,13]. In this context, the higher germination and germination rate observed for the cultivar Fairfax indicates a greater capacity of this genotype to maintain germinative metabolism even under osmotically unfavorable conditions, possibly due to lower water requirements or greater efficiency in reserve mobilization.

The use of growth regulators and other bioactive compounds in the pre-germinative treatment of seeds represents an efficient strategy to mitigate the effects of salinity during the early stages of plant development. This effect was observed in the present study, in which increases in germination and GSI were recorded compared with the saline stress condition, regardless of cultivar. Such treatments may accelerate the germination process, improve seedling vigor, and favor crop establishment, particularly in environments subjected to abiotic stresses [18]. In watermelon, positive effects of gibberellic acid application have already been reported, including improvements in germination and seedling vigor, especially under stress conditions [19]. However, high doses of this regulator may cause phytotoxic effects, highlighting the importance of defining appropriate concentrations for each species and experimental condition [20].

The period between germination and seedling establishment is considered one of the most sensitive to abiotic stresses, as intense physiological processes occur during this phase, including cell division, tissue expansion, and reserve mobilization [21]. Under saline conditions, these processes may be impaired, resulting in reduced growth and delayed early plant development, as observed in both cultivars. According to [13], plant responses to salt stress can be divided into two main phases: an initial phase of growth inhibition associated with osmotic effects and a subsequent phase related to the accumulation of toxic ions and the disruption of cellular metabolism.

Although watermelon is considered a relatively salt-sensitive species, different tolerance levels can be observed among cultivars [3]. The results obtained reinforce this genetic variability, demonstrating greater tolerance of the cultivar Fairfax compared with Crimson Sweet. This superiority may be associated with a greater capacity to maintain seedling growth under stress, possibly related to intrinsic physiological characteristics of the genotype or to greater reserve availability in the seeds, a factor that contributes to early seedling establishment [22,23].

Nevertheless, positive effects of the pre-germinative treatments were also observed for seedling length under salt stress in both cultivars evaluated in this study. In studies with melon, for example, the use of plant regulators such as gibberellic acid and salicylic acid promoted improvements in growth and early seedling development under salinity conditions [20]. These effects may be related to the action of these compounds in activating signaling pathways and inducing physiological and biochemical mechanisms involved in stress tolerance [24,25].

The increase observed in the levels of osmoprotective compounds, such as total soluble sugars, free amino acids, and proline, represents an important mechanism of plant adaptation to salt stress. These metabolites play a fundamental role in cellular osmotic adjustment, contributing to the maintenance of water potential and structural integrity of cells under salinity-induced water deficit conditions [13,15,24]. It was observed that, for total soluble sugars, the treatment with GA resulted in the highest values in both cultivars, particularly in Fairfax, which showed significantly greater accumulation compared with Crimson Sweet. This result suggests that gibberellic acid may stimulate carbohydrate metabolism and reserve mobilization, favoring the synthesis or accumulation of osmotically active sugars in seedlings.

A similar pattern was observed for free amino acids, in which the gibberellic acid treatment also produced the highest values, particularly in the cultivar Fairfax. This increase is frequently associated with intensified metabolism of amino acids related to stress tolerance, including proline, glycine, and citrulline. In cucurbits, citrulline has been identified as an important metabolite associated with cellular protection under abiotic stress conditions, contributing to the scavenging of reactive oxygen species and to the maintenance of cellular metabolism [26].

During salt stress, there is an increase in the production of reactive oxygen species (ROS), such as hydrogen peroxide (H_2_O_2_), as observed in the two watermelon cultivars evaluated. Excessive accumulation of these molecules may lead to oxidative stress, resulting in damage to cellular components, particularly membrane lipids. This process can be evidenced by increased levels of malondialdehyde (MDA), an important indicator of lipid peroxidation. In the results obtained, the saline condition without attenuators showed higher MDA contents in both cultivars, indicating greater oxidative damage and impairment of cellular membrane integrity [17,27].

It is important to highlight that H_2_O_2_ plays a dual role in plant responses to stress, depending on its concentration, origin, and spatiotemporal distribution within the cells. Endogenously produced H_2_O_2_, especially under severe or prolonged stress conditions, tends to accumulate in an uncontrolled manner, leading to oxidative damage and disruption of cellular homeostasis. In contrast, exogenously applied H_2_O_2_ at low concentrations, such as those used in this study, may act as a signaling molecule, triggering acclimation responses and activating antioxidant defense pathways. This controlled exposure can induce a priming effect, enhancing the plant’s ability to cope with subsequent stress conditions [4,9,16].

Conversely, the application of pre-germinative treatments contributed to reducing the oxidative damage caused by salinity. Among the attenuators evaluated, salicylic acid (SA) and hydrogen peroxide (H_2_O_2_) were particularly notable, promoting more pronounced reductions in MDA levels, especially in the cultivar Fairfax. This result reinforces the signaling role of exogenous H_2_O_2_, which, rather than contributing to oxidative damage, likely activated antioxidant enzymes in advance, improving the efficiency of reactive oxygen species scavenging under saline stress. These results indicate that such treatments favored the activation of antioxidant mechanisms in the seedlings, increasing the capacity for ROS scavenging and reducing membrane lipid peroxidation. Thus, the attenuators contributed to maintaining cellular stability under salt stress, with a more pronounced response in the cultivar Fairfax, demonstrating greater physiological efficiency of this genotype in activating defense mechanisms.

To mitigate these effects, plants activate antioxidant defense mechanisms, including enzymatic systems responsible for eliminating these reactive molecules. The results obtained indicate that seed treatment with salicylic acid and hydrogen peroxide stimulated the activity of important antioxidant enzymes, such as catalase (CAT) and ascorbate peroxidase (APX), particularly in the cultivar Fairfax. The increased activity of these enzymes suggests a greater capacity to eliminate hydrogen peroxide generated during stress, thereby contributing to the maintenance of cellular redox homeostasis. According to Zheng et al. [11], the increased activity of antioxidant enzymes may be associated with enhanced H_2_O_2_ production in peroxisomes and the intensification of metabolic processes related to photorespiration under stress conditions.

Salicylic acid plays an important role in the signaling of plant defense responses and can induce the activation of antioxidant enzymes, thereby increasing plant tolerance to different abiotic stresses. The exogenous application of this compound has been associated with increased activity of enzymes such as superoxide dismutase, catalase, and peroxidases, contributing to cellular protection against oxidative damage [25]. Similar results have also been observed in cucurbits, in which seed treatment with salicylic acid promoted greater tolerance to salt stress, reflected in improved seedling performance [28].

Similarly, hydrogen peroxide may act as a signaling molecule when applied at low concentrations, stimulating antioxidant defense mechanisms and contributing to the regulation of redox balance in plant cells [9]. In addition to promoting germination and early seedling development under stress conditions, treatment with H_2_O_2_ may also exert positive effects during later stages of plant development [29]. In watermelon, studies have shown that seeds treated with hydrogen peroxide may produce plants with greater biomass accumulation and improved physiological performance throughout the crop cycle [30].

Overall, the cultivar Fairfax exhibited superior physiological and growth performance under saline conditions, indicating greater tolerance to salt stress. In this cultivar, treatment with SA promoted a more efficient balance between osmotic adjustment and regulation of the antioxidant system, resulting in lower oxidative damage and improved seedling development. This response suggests that osmotic adjustment may represent a primary line of defense in Fairfax, contributing to the maintenance of cellular water status and reducing the intensity of oxidative stress, thereby decreasing the demand for emergency activation of antioxidant mechanisms. This behavior suggests that Fairfax possesses a greater capacity to activate defense mechanisms and to benefit from the stimulus provided by the attenuator, resulting in more vigorous seedlings even under saline conditions.

Conversely, the cultivar Crimson Sweet, considered more sensitive, also responded positively to SA treatment, although less markedly. Although activation of osmotic adjustment and antioxidant defense mechanisms occurred, this cultivar showed greater limitations in growth under stress, indicating lower efficiency in utilizing these mechanisms to sustain seedling development, in this case, the greater accumulation of reactive compounds and activation of compensatory mechanisms suggest that these response are triggered by stress-induced damage rather than preventing it. This suggests a lower efficiency in osmotic adjustment, leading to earlier cellular disturbance and increased reliance on antioxidant defenses Thus, the results suggest that salicylic acid was the most effective attenuator for both cultivars, although the effect was more pronounced in Fairfax due to the greater physiological capacity of this genotype to cope with salt stress.

Similar findings were reported in a recent study conducted with watermelon cultivars subjected to water deficit, in which pre-germinative treatment with stress attenuators promoted greater seedling tolerance through modulation of the antioxidant system and osmotic adjustment. In that study, salt-sensitive watermelon cultivars exhibited greater activation of the enzymatic antioxidant system, whereas more tolerant genotypes demonstrated a greater capacity for osmotic adjustment through the accumulation of compatible solutes. Together, these results reinforce that the coordination between osmotic adjustment and antioxidant defense is genotype-dependent, with tolerant cultivars prioritizing preventive mechanisms, while sensitive cultivars rely more heavily on reactive stress responses. These findings reinforce that stress tolerance mechanisms may vary among cultivars and depend on the predominant physiological strategy of each genotype [19].

Given the increasing salinization of soil in several agricultural regions, the development of strategies that enable the maintenance of crop productivity in saline environments has become essential. In this context, seed treatment with stress-attenuating compounds represents a promising alternative to enhance seedling vigor and stimulate physiological and biochemical mechanisms associated with salinity tolerance, thereby contributing to the successful establishment of watermelon crops under adverse conditions.

## 4. Materials and Methods

The study was conducted at the Seed Analysis Laboratory (LAS) of the Department of Agronomic and Forest Sciences (DCAF) of the Federal Rural University of the Semi-Arid (UFERSA) and at the Plant Physiology and Biochemistry Laboratory (LFBP) of the State University of Rio Grande do Norte (UERN), Mossoró, RN, Brazil.

The experiment was carried out in two stages. Stage I aimed to select two watermelon cultivars (one sensitive and one tolerant to salinity stress) and to determine the salinity level to be used in Stage II. In Stage II, the enzymatic defense system of the species under saline conditions was evaluated, with seeds previously treated with stress attenuators.

### 4.1. Stage I—Selection of Watermelon Cultivars and Determination of the Saline Stress Level to Be Used in Stage II

The experimental design was completely randomized, with four replicates of 50 seeds, arranged in a 3 × 6 factorial scheme. The first factor corresponded to salinity levels (N1 = 0; N2 = −0.2; N3 = −0.4 MPa), while the second factor consisted of six watermelon cultivars (C1 = Crimson Sweet; C2 = Fairfax; C3 = Charleston Gray; C4 = Charleston Super; C5 = Omaru; C6 = Congo).

To simulate salinity stress conditions, seeds from each replicate were placed between three sheets of paper towel moistened with sodium chloride (NaCl) solutions at osmotic potentials of 0, −0.2, and −0.4 MPa [31]. The volume of solution applied to each experimental unit (paper roll) was calculated as 2.5 times the dry mass of the paper used in that replicate [32], following standard procedures for substrate moistening. The paper towel used was Germitest^®^, ensuring uniform moisture e distribution and reproducibility. The rolls were placed in a germination chamber (Lucadema®, LUCA 161/02, São José do Rio Preto – SP, Brazil) at 25 °C.

Evaluations were performed daily by counting the number of germinated seeds up to the fourteenth day after sowing [32]. Seeds that produced normal seedlings were considered germinated. The following variables were analyzed:

Germination (G) and Germination Speed Index (GSI): germination was determined by the percentage of normal seedlings at fourteen days after sowing [32], while the GSI was calculated based on daily counts of normal seedlings [33].

Seedling growth and dry mass accumulation: at the end of the germination test, ten normal seedlings were randomly selected, and shoot length (SL) and primary root length (RL) were measured using a millimeter-graduated ruler. In addition, shoot dry mass (SDM) and root dry mass (RDM) were placed in paper bags and dried in a forced-air circulation oven at 65 °C, followed by weighing on a precision balance (Bel Engineering®, model M214Ai, Via Carlo Carrà 5, Monza, Itália) with precision of 0.0001 g.

The remaining normal seedlings from each replicate were collected and placed in Falcon^®^ tubes, subsequently frozen in liquid nitrogen (−196 °C), and stored in an ultrafreezer (Indrel Scientific®, model Indrel CLC 300DAF, Londrina-PR, Brazil) at −80 °C. The plant material was initially used for the preparation of the crude extract. For this purpose, 0.2 g of fresh seedling mass was weighed and placed in hermetically sealed tubes, to which 3 mL of 60% ethanol was added for automated maceration of the material. The tubes were then placed in a water bath at 60 °C for 20 min and subsequently centrifuged (Novatecnica®, model NT 805, Piracicaba-SP, Brazil) at 10,000 rpm for 10 min at 4 °C. The supernatant was collected to determine the following variables:

Non-enzymatic biochemical determinations: total soluble sugars (TSS) were determined using the Anthrone method [34], with results expressed in mg glucose g^−1^ fresh mass; total free amino acids (TFAA) were determined using the acid ninhydrin method [35], with results expressed in mg glycine g^−1^ fresh mass; and free proline content (PL) was determined according to the method proposed by Bates et al. [36], with results expressed in µmol proline g^−1^ fresh mass.

### 4.2. Stage II—Salt Stress Attenuators and Watermelon Defense System

This stage was conducted in a 2 × 6 factorial scheme, with the first factor consisting of the two cultivars selected in Stage I (one sensitive and one tolerant to salinity), and the second factor corresponding to the combinations between salinity and seed pre-treatment with stress attenuators [T1 = 0.0 MPa (control), T2 = −0.4 MPa (salinity stress), T3 = −0.4 MPa + hydropriming, T4 = −0.4 MPa + gibberellic acid (0.5 mM), T5 = −0.4 MPa + salicylic acid (0.05 mM), and T6 = −0.4 MPa + hydrogen peroxide (10 mM)].

The pre-treatments were applied via controlled seed imbibition on paper substrate. For each treatment, germination paper (Germitest^®^) was moistened with distilled water (hydropriming) or with the respective attenuator solutions, using a volume equivalent to 2.5 times the dry mass of the paper. The seeds were then distributed on the moistened paper and maintained under these conditions for 8 h, allowing gradual imbibition of the solutions. This duration was defined based on the previously established imbibition curve and was standardized for all treatments.

After this period, the seeds from T3 to T6 were transferred to new paper rolls moistened with NaCl solution at −0.4 MPa for the germination test under saline conditions, while seeds from T1 (control) were placed on paper moistened with distilled water (0.0 MPa), and seeds from T2 were directly placed on paper moistened with NaCl solution at −0.4 MPa without pre-treatment.

The exposure period and concentration of each attenuator were determined according to Leite et al. [19]. Initially, an imbibition curve was established for the two materials selected in Stage I, conducted on blotting paper, which determined the hydropriming period of 8 h. At this time, the moisture content of both materials, as well as the remaining time for radicle emission, were uniform.

In addition to the tests performed in Stage I, Stage II included the evaluation of the enzymatic complex of the seedlings. For this purpose, after biometric measurements, the normal seedlings from each replicate were collected and used to obtain the extracts.

For the determination of lipid peroxidation through malondialdehyde (MDA) content and hydrogen peroxide levels, the crude extract was obtained from 200 mg of fresh matter per replicate. The material was disrupted in liquid N_2_ (−196 °C) and subsequently macerated in 2 mL of TCA (0.1% trichloroacetic acid) containing approximately 20% polyvinylpolypyrrolidone (PVPP) for 1 min. After thorough homogenization, the extract was transferred to Eppendorf^®^ tubes and subsequently centrifuged at 10,000 rpm for 5 min.

For the determination of superoxide dismutase, catalase, and ascorbate peroxidase, the crude extract was obtained from 500 mg of fresh matter per replicate, which was disrupted in liquid N_2_ (−196 °C). After the addition of 20% PVPP to the sample, it was homogenized and macerated for 1 min in 100 mM potassium phosphate buffer (pH 7.5) supplemented with 1 mM EDTA (ethylenediaminetetraacetic acid) and 3 mM DTT (dithiothreitol). Subsequently, the extracts were transferred to Eppendorf^®^ tubes and centrifuged at 10,000 rpm for 30 min at 4 °C. All extraction procedures were performed in an ice bath.

After obtaining the extracts, the following determinations were performed:

Hydrogen peroxide (H_2_O_2_): determined according to the method of Alexieva et al. [37], using potassium iodide and spectrophotometric (Bel Engineering®, model UV/VIS Spectrophotometer, Via Carlo Carrà 5, Monza, Itália) reading at a wavelength of 390 nm. Results were expressed in µmol H_2_O_2_ g^−1^ fresh matter.

Lipid peroxidation (MDA): determined according to the method of Heath and Packer [38], using spectrophotometric readings at wavelengths of 535 and 600 nm. Results were expressed in µmol MDA g^−1^ fresh matter.

Superoxide dismutase activity (SOD): quantified according to the methodology of Gianopolitis and Ries [39], using a light bath. Readings were performed in a spectrophotometer at a wavelength of 560 nm. Results were expressed as activity units per minute per gram of fresh matter (U SOD mg^−1^ protein).

Catalase activity (CAT): determined by monitoring the degradation of H_2_O_2_ spectrophotometrically at 240 nm for 1 min [40], with modifications proposed by Azevedo et al. [41]. Enzyme activity was expressed in µmol min^−1^ mg^−1^ protein.

Ascorbate peroxidase activity (APX): determined according to the procedure proposed by Nakano and Asada [42], with readings performed over a 1 min interval at 290 nm, using a molar extinction coefficient of 2.8 mmol^−1^ L cm^−1^. Results were expressed in µmol min^−1^ mg^−1^ protein.

### 4.3. Statistical Analysis

Data were subjected to analysis of variance (ANOVA), and when significant, the Scott–Knott test (*p* ≤ 0.05) was applied to evaluate the effects of cultivars and salinity levels in Stage I. Subsequently, the data were subjected to cluster analysis using the hierarchical method of Ward’s Minimum Variance, with Euclidean distance used as the dissimilarity measure. For this analysis, the free software PAST 4 was used. In Stage II, when ANOVA results were significant, Student’s *t* test and Tukey’s test were applied to evaluate the effects of cultivars and pre-germination treatments, respectively. Statistical analyses were performed using the statistical program SISVAR^®^ [43].

## 5. Conclusions

Salinity compromises germination and seedling vigor in watermelon, with the cultivars Crimson Sweet, Charleston Gray, and Charleston Super being the most sensitive, whereas Fairfax, Omaru, and Congo exhibit greater tolerance to salt stress.

In the cultivar Crimson Sweet, seed treatment with salicylic acid and hydrogen peroxide promotes greater activity of antioxidant enzymes and reduces hydrogen peroxide and malondialdehyde levels, indicating greater efficiency in mitigating oxidative stress. Thus, for salt-sensitive cultivars such as Crimson Sweet, the use of salicylic acid or hydrogen peroxide as seed pre-treatments is recommended.

For the cultivar Fairfax, gibberellic acid enhances osmotic adjustment, while salicylic acid and hydrogen peroxide activate the antioxidant system, reducing oxidative damage and improved seedling performance under salt stress. Therefore, for tolerant cultivars such as Fairfax, seed treatment with gibberellic acid is recommended as the primary strategy, with salicylic acid and hydrogen peroxide as complementary options.

## Figures and Tables

**Table 1:** Germination (G), germination speed index (GSI), root length (RL), shoot dry mass (SDM) and root dry mass (RDM), total soluble sugars (TSS), free amino acids (FAA), and free proline content (PRO) of watermelon (*Citrullus lanatus* (Thunb.) Matsum. & Nakai) cultivars under different salinity levels.

Cultivars	Salinity	G	GSI	RL	SDM	RDM	TSS	FAA	PRO
	MPa	(%)		(cm seedling^−1^)	(mg seedling^−1^)		mg GLU g^−1^ FM	µmol GLY g^−1^ FM	
Crimson Sweet	0	98 aA	16.4 aB	10.4 aB	7.1 aC	3.5 aD	1.2 cA	47.0 cB	0.1 cA
	−0.2	96 aA	14.5 bD	9.7 aB	7.6 aC	3.3 aD	4.4 bB	68.1 bB	1.0 bB
	−0.4	76 bC	10.8 cC	6.4 bB	5.4 bB	3.0 aC	11.4 aC	84.1 aB	2.7 aB
Fairfax	0	98 aA	22.7 aA	13.9 aA	13.3 bB	8.1 aA	1.9 cA	61.0 bA	0.2 cA
	−0.2	98 aA	18.7 bB	12.1 bA	17.5 aA	7.5 aA	5.7 bA	80.3 aA	1.3 bA
	−0.4	95 aA	14.9 cA	10.7 bA	11.8 bA	7.5 aA	12.5 aB	80.9 aB	2.3 aC
Charleston Gray	0	100 aA	23.4 aA	7.8 cC	12.8 bB	5.0 bC	1.8 cA	58.6 cA	0.1 cA
	−0.2	97 aA	18.7 bB	13.6 aA	15.7 aA	6.9 aB	6.0 bA	85.1 bA	1.5 bA
	−0.4	74 bC	11.3 cC	9.3 bA	10.7 cA	4.9 aB	13.4 aA	100.0 aA	3.3 aA
Charleston Super	0	99 aA	23.7 aA	11.4 bB	15.2 aA	6.5 aB	1.4 cA	56.6 cA	0.1 cA
	−0.2	88 bB	20.0 bA	12.9 aA	16.2 aA	6.2 aB	5.0 bB	68.0 bB	0.9 bB
	−0.4	60 cD	12.5 cB	7.1 cB	11.3 bA	4.0 bB	12.4 aB	88.0 aB	3.1 aA
Omaru	0	99 aA	17.2 aB	12.0 aB	12.1 aB	4.4 aD	1.3 cA	47.3 cB	0.1 cA
	−0.2	97 aA	16.3 aC	12.6 aA	13.5 aB	4.9 aC	6.5 bA	68.4 bB	0.8 bB
	−0.4	87 bB	14.2 bA	10.3 bA	11.9 aA	4.3 aB	13.3 aA	87.0 aB	2.2 aC
Congo	0	99 aA	17.5 aB	11.0 bB	12.0 bB	5.4 cC	1.7 cA	50.6 cB	0.2 cA
	−0.2	99 aA	16.2 bC	12.9 aA	14.1 aB	7.9 aA	3.6 bC	72.8 aB	0.9 bB
	−0.4	85 bB	12.3 cB	10.8 bA	9.5 cA	6.6 bA	9.8 aD	64.6 bC	1.8 aD
CV (%)		4.4	4.7	9.4	10.2	12.1	9.9	5.6	15.3

Lowercase letters indicate differences among salinity levels within each cultivar, whereas uppercase letters indicate differences among cultivars within each salinity level, according to the Scott–Knott test at 5% probability. CV = coefficient of variation.

## Data Availability

Data is contained within the article.
